# Hyperuricemia Is Associated With Higher Mortality in Non-diabetic Heart Failure Patients

**DOI:** 10.7759/cureus.75394

**Published:** 2024-12-09

**Authors:** Sergio Madureira, Rita Gouveia, Catarina Elias, Ana Neves, Pedro Ribeirinho-Soares, Marta Amorim, Marta Soares, Joana Pereira, Jorge Almeida, Patrícia Lourenço

**Affiliations:** 1 Department of Internal Medicine, Unidade Local de Saúde de São João, Porto, PRT; 2 Department of Internal Medicine, Centro Hospitalar Póvoa de Varzim - Vila do Conde, Vila do Conde, PRT; 3 Department of Medicine, Faculty of Medicine - University of Porto, Porto, PRT

**Keywords:** biomarker, diabetes mellitus, heart failure, mortality, uric acid

## Abstract

Introduction: Hyperuricemia (HU) is associated with an increased risk of incident heart failure (HF) and adverse HF outcomes. Patients with diabetes mellitus (DM) have a greater prevalence of HU.

Aims: We evaluated the prognostic impact of HU in patients with HF according to the coexistence of DM.

Methods: A retrospective cohort study of ambulatory patients with HF with left ventricular systolic dysfunction (LVSD) was conducted from January 2012 to May 2018. The end point was all-cause mortality; follow-up was until January 2021. A Cox regression analysis was used to assess the prognostic impact of elevated uric acid (UA) levels. The cut-off for HU was 8.2 mg/dL. A multivariate model was built accounting for confounders. The analysis was stratified according to DM, and interaction between DM and UA levels was tested.

Results: We studied 538 patients, of whom 66% were males. Of the patients, 45% had ischemic HF, 41% had DM, and 11% were receiving urate-lowering therapies. The median (interquartile range (IQR)) admission UA was 5.5 (5.8-9.2) mg/dL, and 40% had UA > 8.2 mg/dL. During a median 46-month follow-up, 48.5% of patients died. Patients with UA > 8.2 mg/dL had a multivariate-adjusted hazard ratio (HR) (95% confidence interval (CI)) of all-cause mortality of 1.75 (1.20-2.55; p=0.003). The interaction between DM and UA levels was significant (p=0.04). The independent association of hyperuricemia with mortality persisted only in non-DM patients (HR: 1.70, 95% CI: 1.16-2.51; p=0.007). In those with DM, hyperuricemia portended no survival disadvantage.

Conclusion: DM appears to influence the prognostic impact of HU in chronic HF. The risk of all-cause mortality in hyperuricemic HF patients without DM increases by 70% when compared with those with normal UA levels.

## Introduction

Over the past few decades, several studies reported a correlation between elevated uric acid (UA) levels and cardiovascular disease (CVD) [1,2]. As a result, there is increasing interest in UA as a potential biomarker in heart failure (HF). Hyperuricemia (HU) has been independently associated with increased incidence of HF [1,3] and worse functional outcomes and worse prognosis in patients with established HF, both with reduced and preserved ejection fraction [3-5]. The pathophysiological link between HU and increased risk of CVD, namely, HF, is yet to be established. Serum UA levels are determined by the balance between UA production from purines' metabolism and its renal excretion, and HU commonly results from impaired renal excretion of UA.

Studies addressing the relationship between HU and HF severity show that the negative prognostic impact of HU is independent of concomitant chronic kidney disease or diuretic therapy, suggesting that impaired renal excretion may not be the mechanism behind this association [6]. The rate-limiting step of UA production is the enzymatic reaction of the xanthine dehydrogenase/xanthine oxidase (XO) enzyme that oxidizes hypoxanthine-xanthine into UA. Besides UA production, XO activity also leads to the production of radical oxygen species (ROS). Therefore, increased XO activity leads to increased ROS, promoting an inflammatory reaction that leads to impaired endothelial function [7]. It is still controversial whether HU alone is a causal factor in the inflammatory response [7,8]. Currently, there is limited data regarding whether UA-lowering therapy (ULT) can be used in the prevention or treatment of HF. Although some smaller studies indicate that reducing UA and blocking ROS may have cardioprotective effects [9-11], other studies failed to find any clinical benefits or improved prognosis for patients with HF [12,13].

Patients with diabetes mellitus (DM) have been reported to have increased XO activity and a greater prevalence of HU [14]. HU seems to be associated with increased prevalence and severity of DM microvascular and macrovascular complications [15-18]. This is particularly important because DM is a very prevalent condition in HF patients [19,20], and its coexistence significantly worsens the HF prognosis. The relationship between HF, DM, and HU is even more tangled. Our aim was to assess the impact of HU on the outcome of patients with established HF and determine if the coexistence of DM affects its prognostic value.

This paper was presented as a poster at the Heart Failure Congress that was held in Madrid in 2022.

## Materials and methods

We conducted a retrospective cohort study in ambulatory adult (>18 years old) patients with HF with left ventricular systolic dysfunction (LVSD) (ejection fraction < 50%) followed in a specialized HF clinic of the Internal Medicine department of the Centro Hospitalar e Universitário São João (CHUSJ) from January 2012 to May 2018. CHUSJ is a tertiary care academic hospital in Northern Portugal. The diagnosis of HF was made according to the 2021 European Society of Cardiology guidelines [21]. The echocardiogram for diagnosis establishment was performed peri index visit. For patients referred from the hospital (patients followed in other consultations and patients hospitalized for acute HF), the echocardiograms had been performed recently (approximately three months) before the index visit, and for patients referred from the primary care, an echocardiogram was performed in the index visit or within three months. The index observation was considered the first medical visit of each patient from January 2012 until May 2018. Patients with HF with preserved ejection fraction were excluded from the analysis. Demographic data and data concerning comorbidities, physical examination, echocardiogram, laboratory parameters, and medication at the end of the index visit were recorded.

Hypertension (HTN) was defined as a systolic blood pressure record of ≥140 mmHg and/or diastolic blood pressure of ≥90 mmHg in at least two separate measurements, the presence of previous diagnosis, or record of antihypertensive pharmacological treatment. DM was defined as either a known previous diagnosis, a current prescription of antihyperglycemic agents, or at least two of the following: a fasting venous blood glucose above 126 mg/dL, a random glucose > 200 mg/dL, or a glycated hemoglobin (HbA1c) ≥ 6.5%. Coronary heart disease (CHD) was defined as a history of acute myocardial infarction or a significant CHD image confirmed. Patients were considered to have chronic kidney disease if the index estimated glomerular filtration rate (eGFR) according to the Modification of Diet in Renal Disease (MDRD) formula was <60 mL/minute/m^2^ [22]. Severe left ventricular systolic dysfunction was considered when left ventricular ejection fraction (LVEF) was <30%.

In our hospital, hemoglobin is obtained using a Sysmex-XN-5000® automated blood counter (Sysmex, Matosinhos, Portugal), which utilizes fluorescent flow cytometry and hydrodynamic technologies. Serum creatinine and sodium are measured using conventional methods with an Olympus AU5400® automated clinical chemistry analyzer (Beckman Coulter Diagnostics, Brea, CA), and B-type natriuretic peptide (BNP) determination is also a routine laboratory procedure using a chemiluminescent microparticle immunoassay (two-step immunoassay) (Abbott, Abbott Park, IL). UA is obtained by a colorimetric enzymatic method using an AU2700® automated chemistry analyzer (Beckman Coulter Diagnostics, Brea, CA).

The end point under analysis was all-cause mortality, and vital status was assessed by consulting hospital registries and telephone contact with the patients or their relatives. When no information was obtained, we consulted the Registo Nacional de Utentes (RNU) platform. Patients were followed until January 2021. Follow-up was set since the first medical appointment from 2012 onward. No patient was lost to follow-up.

The study protocol conforms to the ethical guidelines of the Declaration of Helsinki, and it was approved by the CHUSJ Ethics Committee. Due to the retrospective nature of the design, informed consent was waived.

Statistical analysis

Categorical variables are presented as counts and proportions and continuous variables as mean ± standard deviation (SD) when normally distributed or as median (interquartile range (IQR)) when non-normally distributed. The cut-off value used to define hyperuricemia (HU) was 8.2 mg/dL, corresponding to our laboratory's upper limit of the reference range. Patients with UA levels ≤ 8.2 mg/dL and those with higher levels were compared: chi-square test for categorical variables, Student's t-test for normally distributed continuous variables, and the Mann-Whitney U test for continuous variables with a skewed distribution. We used Cox regression analysis to assess the prognostic impact of elevated UA levels. UA was analyzed both as a categorical variable (cut-off of 8.2 mg/dL considered as HU) and a continuous variable (per 1 mg/dL increase in UA). Multivariate models were built based on variables differently distributed in hyperuricemic and non-hyperuricemic patients and on variables known to be mortality-associated in HF patients. Models of increasing complexity were built. A simpler model 1 accounted for age, gender, HTN history, DM, estimated glomerular filtration rate, urate-lowering therapy use, ischemic heart failure, and severity of LVSD. Model 2 accounted for the following confounders: age, gender, HTN history, DM, creatinine clearance, loop diuretic use, thiazide use, urate-lowering therapy use, ischemic HF, NYHA class, and severity of LVSD. Model 3 included variables in model 2 and also hemoglobin, BNP, beta-blocker, and renin-angiotensin system inhibitors. The interaction between DM and UA levels was formally tested in both models: an interaction term between DM and UA (cut-off: 8.2) was included. The analysis was then stratified according to DM coexistence. The p-value considered for statistical significance was 0.05. Data was stored and analyzed using SPSS software version 28.0 (IBM Corp., Armonk, NY).

## Results

We studied 538 patients, of whom 357 (66%) were males. A total of 245 (45.5%) patients had ischemic HF, and 329 (61%) had hypertension (HTN). The median estimated glomerular filtration rate (eGFR) was 54 mL/minute/1.73 m^2^. Of the patients, 11% were receiving urate-lowering therapies, and 446 (83%) were treated with furosemide and 22 (4.1%) with thiazide. Concerning HF disease-modifying drugs, the patients were well-medicated according to updated guidelines by the time of study entry. DM was present in 222 (41.3%) patients; all these patients presented type 2 DM. The median duration of DM was 10 years. Overall, 137 (61.7%) patients were medicated with metformin and 116 (52.3%) with other antihyperglycemic agents (generally dipeptidyl peptidase IV inhibitors or sulfonylureas). Insulin therapy was prescribed to 65 (29.3%) patients.

Patient characteristics and comparison between those with UA levels of 8.2 mg/dL or lower and those with higher levels are depicted in Table 1. The median (IQR) admission UA was 5.5 (5.8-9.2) mg/dL, and 40% had UA levels of >8.2 mg/dL. Patients with HU (UA > 8.2 mg/dL) were older and showed a significantly higher male predominance; they more often presented HTN history, ischemic HF, and a higher prevalence of severe left ventricular systolic dysfunction. Patients with UA levels of >8.2 mg/dL were more symptomatic, and they presented worse renal function and higher natriuretic peptide system activation. More importantly, disease-modifying drugs were similarly prescribed in patients with normal and elevated UA levels. However, loop diuretics were more often prescribed in hyperuricemic patients, and ULT was more often prescribed among those with UA ≤ 8.2 mg/dL. UA levels were similar in patients with and without DM, at 7.6 (5.8-9.2) mg/dL and 7.5 (5.8-9.2) mg/dL, respectively (p=0.68).

**Table 1:** Patient characteristics and comparison between patients with hyperuricemia and the rest BNP: B-type natriuretic peptide, HF: heart failure, IQR: interquartile range, LVSD: left ventricular systolic dysfunction, MRA: mineralocorticoid receptor antagonist, NYHA: New York Heart Association, RASi: renin-angiotensin system inhibitors, SD: standard deviation, UA: uric acid, eGFR: estimated glomerular filtration rate

Characteristic	All patients (N=538)	UA ≤ 8.2 mg/dL (n=328)	UA > 8.2 mg/dL (n=210)	p-value
Age (years), mean (SD)	71 (12)	70 (12)	73 (12)	0.001
Male gender, number (%)	357 (66.4)	202 (61.6)	155 (73.8)	0.003
Arterial hypertension, number (%)	329 (61.2)	186 (56.7)	143 (68.1)	0.008
Diabetes mellitus, number (%)	222 (41.3)	130 (39.6)	92 (43.8)	0.34
Ischemic HF, number (%)	245 (45.5)	137 (41.8)	108 (51.4)	0.03
Severe LVSD, number (%)	251 (46.7)	134 (40.9)	117 (55.7)	<0.001
NYHA class, number (%) (I, II, III/IV)	188 (34.9), 235 (43.7), 115 (21.4)	138 (42.1), 138 (42.1), 52 (15.9)	50 (23.8), 97 (46.2), 63 (30.0)	<0.001
Uric acid (mg/dL), median (IQR)	7.5 (5.8-9.2)	-	-	-
Hemoglobin (g/dL), mean (SD)	13.2 (1.8)	13.2 (1.8)	13.0 (2.0)	0.21
eGFR (mL/minute/1.73 m^2^), median (IQR)	54 (40-71)	61 (48-79)	43 (32-57)	<0.001
BNP (pg/mL), median (IQR)	251.9 (113.0-637.9)	250.9 (103.9-501.6)	350.8 (150.2-835.3)	<0.001
RASi, number (%)	458 (85.1)	286 (87.2)	172 (81.9)	0.10
MRA, number (%)	161 (29.9)	101 (30.8)	60 (28.6)	0.58
Beta-blockers, number (%)	500 (92.9)	300 (91.5)	200 (95.2)	0.10
Loop diuretics, number (%)	446 (82.9)	252 (76.8)	194 (92.4)	<0.001
Thiazide diuretics, number (%)	22 (4.1)	10 (3.0)	12 (5.7)	0.13
Urate-lowering therapy, number (%)	57 (10.6)	45 (13.7)	12 (5.7)	0.003
Follow-up (months), median (IQR)	46 (30-83)	55 (34-88)	39 (24-68)	<0.001>
Mortality, number (%)	261 (48.5)	136 (41.5)	125 (59.5)	<0.001

**Figure 1:**
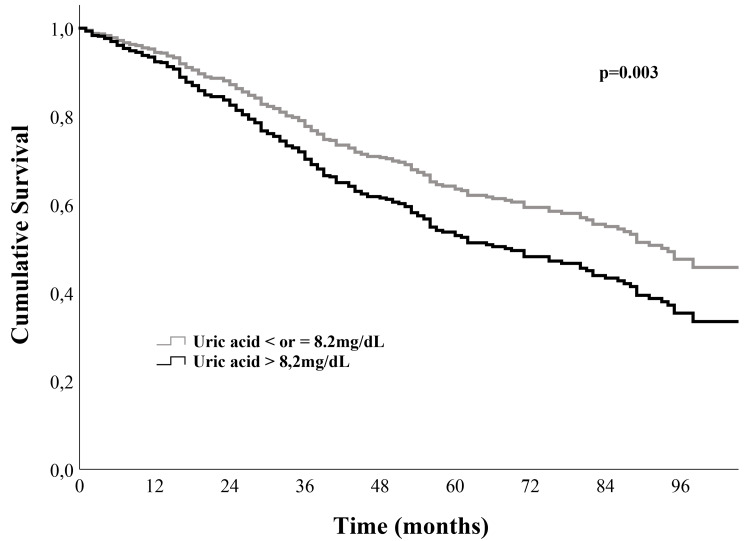
Survival curves according to uric acid level: ≤8.2 mg/dL and > 8.2 mg/dL

During a median follow-up of 46 (IQR: 30-83) months, 261 (48.5%) patients died: 41.5% in non-hyperuricemic patients and 59.5% in hyperuricemic patients (p<0.001). Patients with UA levels of >8.2 mg/dL had a crude hazard ratio (HR) of death of 1.81 (95% confidence interval (CI): 1.42-2.30; p<0.001). After multivariate adjustment for confounding variables, the association of HU with all-cause mortality was 1.94, 1.76, and 1.75 in models 1, 2, and 3, respectively. Figure 1 shows the adjusted (model 3) survival curves for patients with UA levels of ≤8.2 and those with UA levels of >8.2 mg/dL.

When the analysis was stratified according to DM status, the independent association of HU with mortality was only observed in the group of non-DM patients (HR: 2.03, 1.84, and 1.70 in models 1, 2, and 3, respectively), and in the group of HF patients with DM, no independent association was identified. The p-value for interaction between HU and DM was 0.03 in model 1, 0.03 in model 2, and 0.04 in model 3. Table 2 shows the crude and multivariable-adjusted association of HU with all-cause mortality in the whole group of patients and separately in subgroups of patients with and without DM.

**Table 2:** Association of hyperuricemia (uric acid > 8.2 mg/dL) with mortality: crude and multivariate-adjusted Cox regression analysis and stratification according to the coexistence of diabetes mellitus ⁋Model 1: adjustments to age gender, arterial hypertension history, estimated glomerular filtration rate, urate-lowering therapy use, ischemic heart failure, and severity of LVSD. ¥Model 2: adjustments to age, gender, arterial hypertension history, estimated glomerular filtration rate, loop diuretic use, thiazide use, urate-lowering therapy use, ischemic heart failure, New York Heart Association class, and severity of LVSD. For the whole group diabetes mellitus, an interaction term between hyperuricemia and diabetes was also included. §Model 3: variables in model 1 + B-type natriuretic peptide, hemoglobin, beta-blocker use, and renin-angiotensin inhibitor use. For the whole group diabetes mellitus, an interaction term between hyperuricemia and diabetes was also included. UA was analyzed as a categorical variable: p for interaction between hyperuricemia and diabetes mellitus in model 1 = 0.03, model 2 = 0.03, and model 3 = 0.04. CI: confidence interval, DM: diabetes mellitus, HR: hazard ratio, LVSD: left ventricular systolic dysfunction, UA: uric acid

UA > 8.2 mg/dL	All patients (N=538)		Patients with no DM (n=316)		Patients with DM (n=222)	
	HR (95% CI)	p	HR (95% CI)	p	HR (95% CI)	p
Crude	1.81 (1.42-2.30)	<0.001	2.08 (1.48-2.92)	<0.001	1.49 (1.04-2.12)	0.03
Multivariable adjusted - model 1⁋	1.94 (1.35-2.78)	<0.001	2.03 (1.38-2,97)	<0.001	1.08 (0.71-1.63)	0.72
Multivariable adjusted - model 2¥	1.76 (1.23-2.53)	0.002	1.84 (1.25-2.70)	0.002	0.96 (0.63-1.46)	0.84
Multivariable adjusted - model 3§	1.75 (1.20-2.55)	0.003	1.70 (1.16-2.51)	0.007	1.06 (0.68-1.64)	0.81

**Figure 2:**
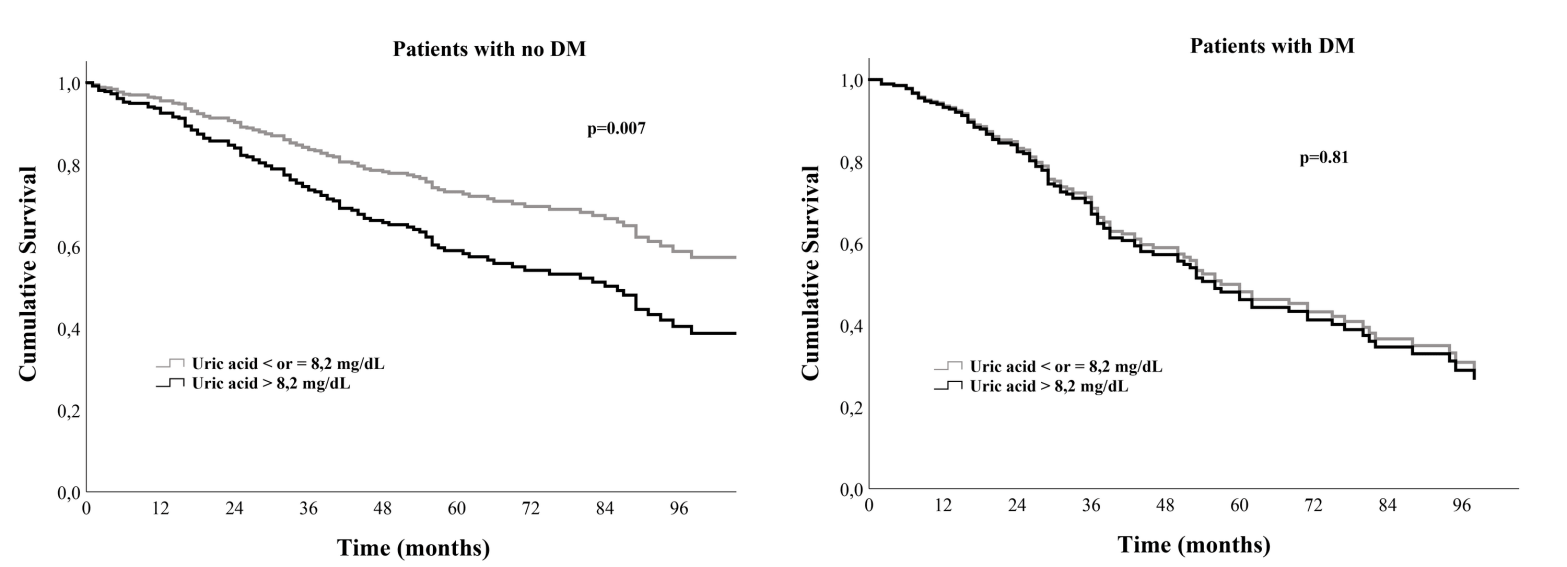
Survival curves according to uric acid level Curves as presented separately in non-diabetic HF patients (left) and HF patients with diabetes mellitus (right) DM: diabetes mellitus, HF: heart failure

Figure 2 shows the adjusted (model 2) survival curves according to the UA cut-off used separately in patients with no concomitant DM (left) and those with DM (right). HU was an independent predictor of all-cause death only in HF patients with no concomitant DM.

If UA was analyzed as a continuous variable, in HF non-DM patients, the HR (95% CI) per 1 mg/dL increase in UA was 1.18 (1.09-1.28), 1.16 (1.07-1.26), and 1.10 (1.02-1.19) in models 1, 2, and 3, respectively (Table 3). No significant prognostic association was present in DM patients.

**Table 3:** Association of uric acid with mortality: crude and multivariate-adjusted Cox regression analysis and stratification according to the coexistence of diabetes mellitus ⁋Model 1: adjustments to age gender, arterial hypertension history, estimated glomerular filtration rate, urate-lowering therapy use, ischemic heart failure, and severity of LVSD. ¥Model 2: adjustments to age, gender, arterial hypertension history, creatinine clearance, loop diuretic use, thiazide use, urate-lowering therapy use, ischemic heart failure, New York Heart Association class, and severity of LVSD. For the whole group diabetes mellitus, an interaction term between hyperuricemia and diabetes was also included. §Model 3: variables in model 1 + B-type natriuretic peptide, hemoglobin, beta-blocker use, and renin-angiotensin inhibitor use. For the whole group diabetes mellitus, an interaction term between hyperuricemia and diabetes was also included. UA was analyzed as a continuous variable: p for interaction between uric acid and diabetes mellitus in model 1 = 0.02, model 2 = 0.03, and model 3 = 0.03. CI: confidence interval, DM: diabetes mellitus, HR: hazard ratio, LVSD: left ventricular systolic dysfunction, UA: uric acid

UA per each 1 mg/dL	All patients (N=538)		Patients with no DM (n=316)		Patients with DM (n=222)	
	HR (95% CI)	p	HR (95% CI)	P	HR (95% CI)	p
Crude	1.16 (1.10-1.22)	<0.001	1.20 (1.12-1.28)	<0.001	1.11 (1.03-1,20)	0.008
Multivariable adjusted - model 1⁋	1.16 (1.08-1.25)	<0.001	1.18 (1.09-1.28)	<0.001	1.10 (0.91-1.12)	0.84
Multivariable adjusted - model 1¥	1.13 (1.05-1.22)	0.001	1.16 (1.07-1.26)	<0.001	0.98 (0.88-1.08)	0.65
Multivariable adjusted - model 2§	1.11 (1.03-1.20)	0.008	1.10 (1.02-1.92)	0.02	0.99 (0.90-1.10)	0.87

## Discussion

In our cohort of over 500 chronic ambulatory HF patients with left ventricular systolic dysfunction, the negative prognostic value of HU was reproducible with higher overall mortality being observed in hyperuricemic patients. However, when dividing our cohort according to concomitant DM or absence of DM, the independent association with all-cause mortality was only valid for HF patients with no concomitant DM. In patients without DM, the presence of HU predicted a 70% higher risk of all-cause mortality in the long term when compared with patients with lower UA levels, and the rise in risk was 10% per each 1 mg/dL increase in UA levels. In HF patients with DM, no independent association with mortality was observed. Our results support the negative impact of HU in HF and suggest that it may be exclusive to non-DM patients.

It is important to remember the lack of a universally accepted definition of HU and the uncertainty concerning the value beyond which the CVD risk increases [5]. The commonly used physicochemical definition corresponds to urate concentrations above 7 mg/dL, measured by automated enzymatic methods, reflecting the urate solubility limit in body fluids [23]. Our laboratory uses a colorimetric method, whose results are approximately 1 mg/dL higher than those obtained with automated methods [24]; this can explain the higher cut-off for HU (>8.2 mg/dL) established by our laboratory.

It has been described that the deleterious cardiovascular effects of elevated UA levels may occur at concentrations below saturation point [25,26], suggesting a role for XO activation, ROS formation, and a pro-inflammatory state as contributors to CVD establishment and progression per se separately from the crystal deposition found in gout [27]. Several studies have tried to establish the optimal cut-off value for UA as a risk marker for CVD, but consensus still lacks [28]. Therefore, we decided to follow the classical, and most used, definition of HU and use the cut-off established by our laboratory.

HF and DM share pro-inflammatory mechanisms in their pathogenesis, and the metabolism of UA may play a role in this inflammatory cascade [17]. Regarding HF, both with reduced and preserved ejection fraction, there is a correlation between elevated serum pro-inflammatory cytokines and adverse clinical outcomes [29]. The pro-inflammatory cytokines found in chronic HF are significantly less elevated than what is observed in classical inflammatory diseases [30], suggesting that low-grade chronic inflammation may contribute to clinical deterioration in patients with established HF. In HF patients, increased UA may result from XO upregulation and reduced renal clearance [31]; additionally, the concurrent use of loop diuretics also contributes to UA rise. It is thought that HU's negative prognostic role comes from inducing inflammation and the formation of ROS with consequent endothelial damage [27,31].

DM is also a pro-inflammatory condition, and increased XO activity has been reported in diabetic patients [16]. When considering DM patients with HF, higher XO activity and higher UA levels were expected to account for synergic oxidative damage and thus higher mortality. While HU has been linked with DM complications and disease severity [16,18], the diabetic population with HU in our study failed to show an increased mortality. The differential impact of HU on the outcome of HF patients with and without DM was already described in acute HF and is counterintuitive and puzzling [32]. A possible explanation is the fact that DM is a strong enough negative prognostic predictor that overcomes the impact of HU. Blocking ROS accumulation by inhibiting XO has been hypothesized as a promising new approach to HU and CVD risk. However, studies on HF have reached contradictory outcomes. While some studies with allopurinol showed improvement in myocardial function, cardiac fibrosis, and endothelial function [9,10], a recent meta-analysis failed to show a significant mortality risk reduction or improved clinical outcomes [12]. One hypothesis that may explain the conflicting results found is that the trials were conducted in non-selected HF populations. When taking our study into account, non-diabetic HF patients with HU show a higher risk of adverse clinical outcomes when compared to patients with concurring DM. Therefore, we question if XO inhibition and HU reduction could have been beneficial only in the subgroup of non-diabetic patients. This may mask the possible positive impact of ULT on the non-selected HF populations studied in the trials so far published.

Our study has some limitations that should be remembered. It is single-centered, and patients are all followed in a specialized HF clinic of a central tertiary care academic hospital posing concerns regarding the generalizability of the results. Additionally, only patients with left ventricular systolic dysfunction were studied, and therefore, no conclusions can be drawn concerning patients with HF with preserved ejection fraction in whom inflammation is thought to play an even more important role. The retrospective design has inherent setbacks regarding data availability and quality. Adjustments to parameters such as C-reactive protein would have been interesting; however, it is not routinely performed in stable patients. Moreover, knowledge of other inflammatory biomarkers such as tumor necrosis factor-α (TNF-α) or interleukin-6 (IL-6) would be even more interesting. Also, we have only one UA assessment at study entry, and it would have been important to analyze the impact of UA dynamics over time. Finally, despite being a contemporary HF patient population, adjustments to angiotensin receptor neprilysin inhibitors and sodium-glucose cotransporter 2 (SGLT2) inhibitors (iSLGT2) were not possible, first because the study was conducted before their co-payment by the Portuguese health system and the second because it was conducted before the approval of iSGLT2 for the treatment of HF. iSLGT2 have recently shown to lower mortality in HF [33] and also to reduce UA levels in HU patients [34] and are currently widely prescribed both in DM and HF. It would be interesting to reproduce our results in a population of HF patients medicated with iSGLT2. Despite all these limitations, we analyzed a large enough number of events and were able to adjust for multiple confounders. The fact that the simpler model 1 produced results similar to those of more complex models 2 and 3 reinforces the validity of our findings since simpler models are less prone to overfitting and collinearity.

## Conclusions

DM coexistence appears to influence the prognostic impact of hyperuricemia in chronic HF. The risk of all-cause mortality in hyperuricemic HF patients without DM increased by 70% when compared with those with normal UA levels, and the rise in risk was 10% per each 1 mg/dL increase in UA levels. HU showed no prognostic value in diabetic HF patients.

Our results raise the question of whether xanthine oxidase inhibition and uric acid reduction could be beneficial in the subgroup of non-diabetic patients.

## References

[1] Krishnan E (2009). Hyperuricemia and incident heart failure. Circ Heart Fail.

[2] Roger VL (2021). Epidemiology of heart failure: a contemporary perspective. Circ Res.

[3] Krishnan E (2012). Gout and the risk for incident heart failure and systolic dysfunction. BMJ Open.

[4] Chen JH, Chuang SY, Chen HJ, Yeh WT, Pan WH (2009). Serum uric acid level as an independent risk factor for all-cause, cardiovascular, and ischemic stroke mortality: a Chinese cohort study. Arthritis Rheum.

[5] Freilich M, Arredondo A, Zonnoor SL, McFarlane IM (2022). Elevated serum uric acid and cardiovascular disease: a review and potential therapeutic interventions. Cureus.

[6] Borghi C, Cosentino ER, Rinaldi ER, Cicero AF (2014). Uricaemia and ejection fraction in elderly heart failure outpatients. Eur J Clin Invest.

[7] Sharaf El Din UA, Salem MM, Abdulazim DO (2017). Uric acid in the pathogenesis of metabolic, renal, and cardiovascular diseases: a review. J Adv Res.

[8] Mercuro G, Vitale C, Cerquetani E, Zoncu S, Deidda M, Fini M, Rosano GM (2004). Effect of hyperuricemia upon endothelial function in patients at increased cardiovascular risk. Am J Cardiol.

[9] Jia N, Dong P, Ye Y, Qian C, Dai Q (2012). Allopurinol attenuates oxidative stress and cardiac fibrosis in angiotensin II-induced cardiac diastolic dysfunction. Cardiovasc Ther.

[10] George J, Carr E, Davies J, Belch JJ, Struthers A (2006). High-dose allopurinol improves endothelial function by profoundly reducing vascular oxidative stress and not by lowering uric acid. Circulation.

[11] Nishino M, Egami Y, Kawanami S (2022). Lowering uric acid may improve prognosis in patients with hyperuricemia and heart failure with preserved ejection fraction. J Am Heart Assoc.

[12] Kodama S, Fujihara K, Horikawa C (2021). Network meta-analysis of drug therapies for lowering uric acid and mortality risk in patients with heart failure. Cardiovasc Drugs Ther.

[13] Givertz MM, Anstrom KJ, Redfield MM (2015). Effects of xanthine oxidase inhibition in hyperuricemic heart failure patients: the Xanthine Oxidase Inhibition for Hyperuricemic Heart Failure Patients (EXACT-HF) study. Circulation.

[14] Gherghina ME, Peride I, Tiglis M, Neagu TP, Niculae A, Checherita IA (2022). Uric acid and oxidative stress-relationship with cardiovascular, metabolic, and renal impairment. Int J Mol Sci.

[15] Li X, Meng X, Gao X (2018). Elevated serum xanthine oxidase activity is associated with the development of type 2 diabetes: a prospective cohort study. Diabetes Care.

[16] Miric DJ, Kisic BM, Filipovic-Danic S, Grbic R, Dragojevic I, Miric MB, Puhalo-Sladoje D (2016). Xanthine oxidase activity in type 2 diabetes mellitus patients with and without diabetic peripheral neuropathy. J Diabetes Res.

[17] Arersa KK, Wondimnew T, Welde M, Husen TM (2020). Prevalence and determinants of hyperuricemia in type 2 diabetes mellitus patients attending Jimma Medical Center, Southwestern Ethiopia, 2019. Diabetes Metab Syndr Obes.

[18] Kushiyama A, Tanaka K, Hara S, Kawazu S (2014). Linking uric acid metabolism to diabetic complications. World J Diabetes.

[19] Nichols GA, Gullion CM, Koro CE, Ephross SA, Brown JB (2004). The incidence of congestive heart failure in type 2 diabetes: an update. Diabetes Care.

[20] Kannel WB, McGee DL (1979). Diabetes and cardiovascular disease. The Framingham study. JAMA.

[21] McDonagh TA, Metra M, Adamo M (2021). 2021 ESC guidelines for the diagnosis and treatment of acute and chronic heart failure. Eur Heart J.

[22] Levey AS, Bosch JP, Lewis JB, Greene T, Rogers N, Roth D (1999). A more accurate method to estimate glomerular filtration rate from serum creatinine: a new prediction equation. Modification of Diet in Renal Disease Study Group. Ann Intern Med.

[23] Bardin T, Richette P (2014). Definition of hyperuricemia and gouty conditions. Curr Opin Rheumatol.

[24] Barr WG (1990). Chapter 165: Uric acid. Clinical methods: the history, physical, and laboratory examinations, third edition.

[25] Desideri G, Castaldo G, Lombardi A (2014). Is it time to revise the normal range of serum uric acid levels?. Eur Rev Med Pharmacol Sci.

[26] Masulli M, D'Elia L, Angeli F (2022). Serum uric acid levels threshold for mortality in diabetic individuals: the URic acid Right for heArt Health (URRAH) project. Nutr Metab Cardiovasc Dis.

[27] Johnson RJ, Bakris GL, Borghi C (2018). Hyperuricemia, acute and chronic kidney disease, hypertension, and cardiovascular disease: report of a scientific workshop organized by the National Kidney Foundation. Am J Kidney Dis.

[28] Borghi C, Tykarski A, Widecka K (2018). Expert consensus for the diagnosis and treatment of patient with hyperuricemia and high cardiovascular risk. Cardiol J.

[29] Vasan RS, Sullivan LM, Roubenoff R (2003). Inflammatory markers and risk of heart failure in elderly subjects without prior myocardial infarction: the Framingham Heart Study. Circulation.

[30] Mann DL (2015). Innate immunity and the failing heart: the cytokine hypothesis revisited. Circ Res.

[31] Kumrić M, Borovac JA, Kurir TT, Božić J (2021). Clinical implications of uric acid in heart failure: a comprehensive review. Life (Basel).

[32] Cidade-Rodrigues C, Cunha FM, Elias C, Oliveira D, Bettencourt P, Lourenço P (2021). The prognostic impact of uric acid in acute heart failure according to coexistence of diabetes mellitus. Nutr Metab Cardiovasc Dis.

[33] Zannad F, Ferreira JP, Pocock SJ (2020). SGLT2 inhibitors in patients with heart failure with reduced ejection fraction: a meta-analysis of the EMPEROR-Reduced and DAPA-HF trials. Lancet.

[34] Zhao Y, Xu L, Tian D, Xia P, Zheng H, Wang L, Chen L (2018). Effects of sodium-glucose co-transporter 2 (SGLT2) inhibitors on serum uric acid level: a meta-analysis of randomized controlled trials. Diabetes Obes Metab.

